# Two-Directional Tuning of Distributed Feedback Film Dye Laser Devices

**DOI:** 10.3390/mi8120362

**Published:** 2017-12-16

**Authors:** Hongtao Feng, Weiliang Shu, Hong Xu, Baoyue Zhang, Bin Huang, Jingjing Wang, Wei Jin, Yan Chen

**Affiliations:** Shenzhen Institutes of Advanced Technology, Chinese Academy of Sciences, 1068 Xueyuan Ave, Shenzhen 518055, China; ht.feng@siat.ac.cn (H.F.); wl.shu@siat.ac.cn (W.S.); hong.xu@siat.ac.cn (H.X.); by.zhang@siat.ac.cn (B.Z.); bin.huang@siat.ac.cn (B.H.); jj.wang@siat.ac.cn (J.W.); wei.jin@siat.ac.cn (W.J.)

**Keywords:** dye laser, tunable, distributed feedback

## Abstract

We demonstrate a two-directional tuning method of distributed feedback (DFB) film dye laser devices to achieve high quality lasing and a large tuning range. In this work, we proposed a simple method to fabricate a continuous tunable solid-state dye laser on a flexible Polydimethylsiloxane (PDMS) film. In order to obtain stable and tunable output lasing, the stretching property of the gelatine host was improved by mixing with a certain ratio of glycerol to prevent DFB cavity destruction. We employed two different tuning strategies of the DFB film dye lasers, by stretching the PDMS film in two perpendicular directions, and a nearly 40 nm tuning range in each direction was achieved. The laser device maintained single mode lasing with 0.12 nm linewidth during the tuning process. The reported tunable DFB film dye laser devices have huge potential as coherent light sources for sensing and spectroscopy applications.

## 1. Introduction

In recent years, flexible and tunable optical elements have attracted extensive interests in the emerging field of integrated optics in lab-on-a-chip applications [1,2,3]. Organic dye lasers have huge potential to serve as coherent visible light sources in lab-on-a-chip systems, since the organic materials have a broad gain spectrum and offer flexible tuning capabilities, which is favorable in spectroscopy and biosensing applications [4]. Among various types of laser cavity structures, including Fabry-Pérot cavities [5,6], ring resonators [7,8] and distributed feedback (DFB) gratings [9,10,11,12], DFB resonator configuration was widely chosen for its high quality lasing performance, low threshold, and stable operation. In particular, second order DFB lasers have the advantages of surface emission and less optical losses of low order Bragg gratings [13]. The output wavelength λ_laser_ of DFB laser fulfills the Bragg equation λ_laser_ = 2Λn_eff_/m, where n_eff_ is the effective refractive index (RI) of the laser mode, Λ is the period of grating, and m is the Bragg order [14]. According to the equation, the output wavelength of a DFB laser can realize tuning by changing the effective refractive index or the grating period.

Adjusting the grating period is the most convenient and effective way to tune the lasing wavelength of the solid-state DFB lasers. Various methods have been demonstrated, including changing the substrates with different grating periods, fabricating multiple periods of gratings on different locations, bending the grating substrates, and mechanically stretching the grating. Changing different grating substrates or selecting different grating areas on the same substrate required additional costs to prepare the samples, and it is not convenient to integrate in lab-on-a-chip systems [15,16,17]. Kim et al. proposed a tunable DFB laser with 12 nm tuning range by bending the dye laser film, and the emission peak split into two peaks for large bending radius [18]. Suzuki et al. employed an elastomer PVA as the tunable grating layer to construct the DFB laser device, and the tuning range was limited to only 4 nm due to the mechanical property of PVA [19]. Weinberger et al. synthesized a new elastomer material, offering the possibility of large scale mechanical deformation. The fabricated DFB laser achieved a 25 nm tuning range, with relatively strong photoluminescence in the lasing emission [20]. Doring et al. demonstrated a tunable laser with a 47 nm tuning range by voltage induced deformation of electro-active substrate [21]. However, the output lasing with 0.8 nm linewidth exhibited strong photoluminescence and multiple modes, and the relationship of wavelength shift and voltage change was nonlinear. Therefore, it is necessary to develop a stable and linear tuning method of solid-state dye lasers with a large tuning range.

Polydimethylsiloxane (PDMS) is an insulating, light weight and inexpensive elastomer with mechanical deformation up to 120% [22]. Due to its easy fabrication and excellent mechanical property, PDMS was widely used in lab-on-a-chip optical systems. Stretchable optical waveguides entirely fabricated with PDMS were proposed by Missinne et al., substituting traditional rigid materials for optical components [2]. High RI PDMS core material was capillary-filled into a microfluidic channel made by low RI PDMS as the cladding, and the waveguide exhibited excellent optical performance. PDMS film provides superior mechanical properties for tunable optical components. Therefore, PDMS film could be chosen as the flexible substrate for tunable laser devices, and has the potential to provide good lasing performance during repeatedly tuning experiments.

In this work, we developed a new two-directional tuning strategy to continuously adjust the wavelength of DFB dye laser devices. The second-order DFB dye laser was composed of a flexible PDMS film and a gelatine matrix doped with active gain material. To improve the stretching property of gelatine, a certain ratio of glycerol was mixed into the matrix to prevent DFB cavity destruction caused by mechanical deformation. During the tuning process, the dye laser device maintained stable single mode operation with high quality lasing performance. Our simple method to construct a DFB film dye laser provided the opportunity for low-cost mass production of tunable laser devices, and presents high potential in sensing and spectroscopy applications.

## 2. Materials and Methods

### 2.1. Device Structure

**Figure 1:**
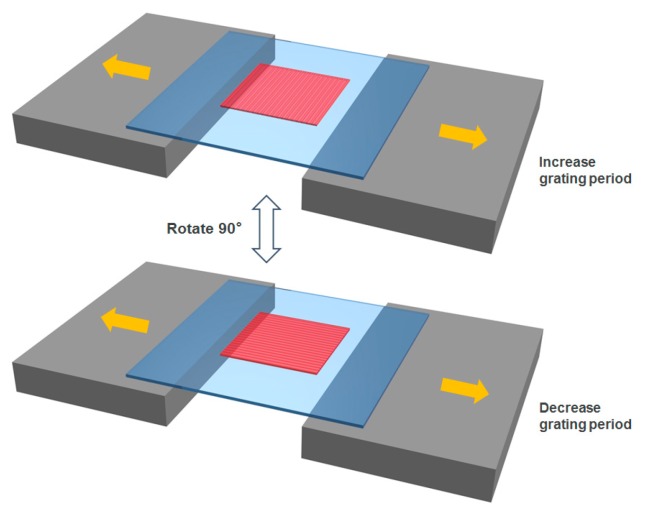
Schematic design of the tunable DFB film dye laser device and the two-directional tuning strategy. The laser wavelength is adjusted by stretching the grating in two directions.

The DFB dye laser was constructed on a flexible PDMS film by replica molding process. One-dimensional grating structures with 417 nm period and 100 nm depth was employed as the corrugation patterns. The active layer composed of a gelatine host doped with Rhodamine 6 G or Rhodamine 101 was spin-coated on the PDMS grating thin film. The emission spectra of the laser dyes cover a broad spectrum in the visible range. Sub-micrometer thickness gelatine film (RI = 1.55) on top of PDMS cladding layer (RI = 1.406) with grating structures formed the laser resonator. By modifying the components in the active layer, the PDMS-based matrix could be stretched back and forth repeatedly, achieving laser tuning in a wide range without surface cracking and wrinkles. The DFB film dye laser was stretched in two perpendicular directions, providing two different laser tuning modes, as shown in Figure 1. The wavelength of the output laser could increase by stretching the film perpendicular to the grating, or decrease by stretching the film parallel to the grating.

### 2.2. Fabrication

**Figure 2:**
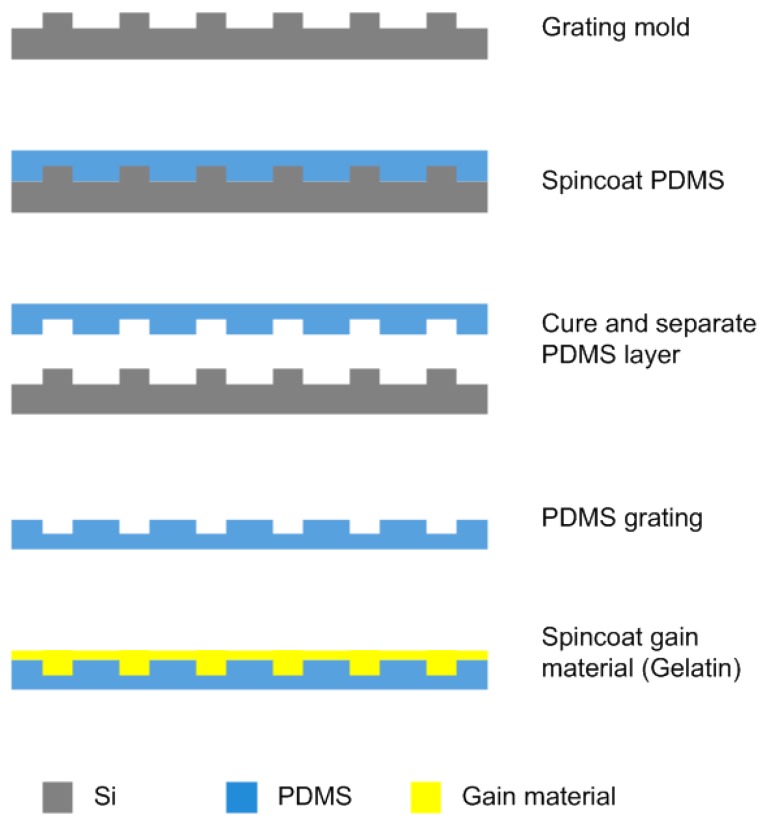
The schematic fabrication process of the tunable DFB film dye laser device.

The schematic fabrication process of the PDMS film dye laser device is shown in Figure 2. The flexible film dye laser device was made by a simple replica molding process. The grating mold used for replica molding was fabricated by nanoimprint lithography [23,24]. After pattern transfer, the grating mold was etched to 100 nm depth using reactive ion etching (RIE-10, Samco, Japan). Next, the PDMS prepolymer (RTV 615, General Electric, 1:10 ratio) was mixed and poured on the grating mold coated with an anti-sticking agent (Trimethychlorosilane (TMCS), ≥98%) in vapor phase. The uniform film layer with 1 mm thickness was made by controlling the total weight of PDMS. Then the material was placed under vacuum for 30 mins to ensure pattern transfer fidelity, and baked at 80 °C overnight. After separating the PDMS layer with the grating mold, the surface was exposed to oxygen plasma for 30 s to improve adhesion between the cladding and active layers.

Next, the active layer was made by dissolving gelatine in water at a concentration of 6.8% (*w*/*w*) at 65 °C and stirring for 2 h, and then glycerol was added to the mixture at a concentration of 5% (*w*/*w*) to prevent surface cracking. Rhodamine 6 G (RH 6 G) and Rhodamine 610 (RH 610) were added separately to the hot gelatine matrix as the gain materials. The concentration of the gain medium used in our film dye laser device was typically 1 mg/mL. The hot dye/polymer solution (65 °C) was immediately spin-coated onto the grating substrate at 3000 rpm for 60 s, in order to avoid surface roughness caused by low-temperature gelatine coagulation, and generate a 250 nm thick homogeneous active layer.

## 3. Results and Discussion

### 3.1. Laser Characterization

**Figure 3:**
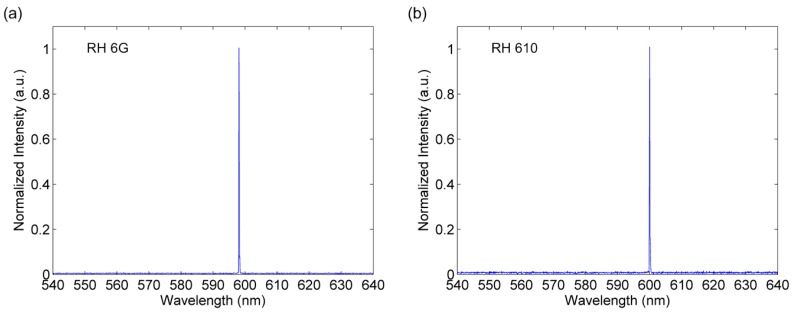
Typical emission spectra of DFB film dye lasers doped with (**a**) RH 6 G and (**b**) RH 610.

The optical performance of the tunable DFB film dye laser device is analyzed by measuring quality factors and lasing thresholds. The DFB film dye laser was optically pumped with a 10 ns 532 nm Nd:YAG laser (Elforlight, SPOT 10-200-532, Daventry, Northants, UK). The excitation frequency was triggered by an external signal generator at 1 Hz and the input energy was adjusted by an optical density filter plate. A 10× objective was used to focus input light on the surface of PDMS film, with the spot size of 777 μm. The output laser was collected by a 4× microscope objective from the top surface of the chip and delivered to a fiber coupled spectrometer (Ocean Optics, HR2000+, Largo, FL, USA) with 0.1 nm resolution. In order to fully detect output laser intensity, the integration time was set to 1 s to synchronize with excitation frequency. In order to achieve a wide laser wavelength tuning range, we chose two laser dyes, RH 610 and RH 6 G, to cover nearly 100 nm spectrum from 550 to 650 nm, since the emission spectrum range of a single laser dye is typically 30–50 nm. The typical lasing spectra for dye lasers doped with RH 6 G and RH 610 are shown in Figure 3, with output laser wavelength of 598.15–600.04 nm. The full width at half maximum (FWMH) of the lasers were both measured to be 0.12 nm. The wavelengths were consistent with the theoretical calculation, which satisfied the Bragg resonance condition formula λ_laser_ = 2Λn_eff_/m. Due to the variation of the film thickness, there was a slight difference between individual film laser devices.

**Figure 4:**
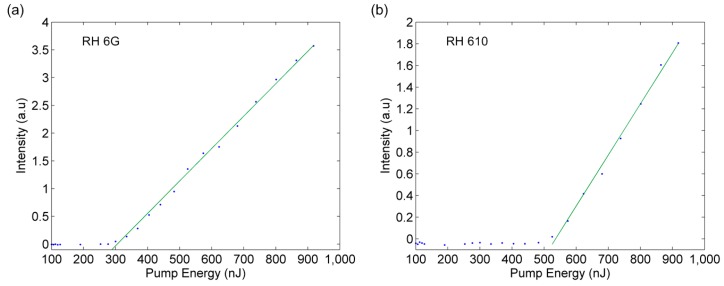
The input–output curve of DFB film dye lasers doped with (**a**) RH 6 G and (**b**) RH 610, showing the lasing threshold energy.

The threshold pump energy of the PDMS film lasers with RH 6 G and RH 610 were measured. By adjusting the optical density filter, we could tune the input laser intensity and measured the output laser intensities. The plots of laser output vs. pumping energy of DFB lasers with two gain materials are shown in Figure 4. The absorbed pump threshold energy of two dye lasers upon excitation were 304.97–535.51 nJ, respectively. The threshold powers were estimated to be 642.18–1.129 μJ/mm^2^ by linear fitting. The difference in threshold energy is due to the different photoluminescence efficiency of the dyes under the same excitation condition. The measured output pulse energies were 19.8–10 nJ at a maximum input energy of 918 nJ using a pulse-energy meter, and the efficiency of the lasers were approximately 2.2% and 1.1%, respectively.

### 3.2. Laser Wavelength Tuning

Various methods such as changing the n_eff_, period, or *m* can be used to tuned the lasing wavelength, due to the low Young's modulus (~750 kpa) of PDMS, we employed a straight forward method to change the grating period of the dye lasers by stretching the PDMS flexible film. We have developed a two-directional lasing wavelength tuning scheme by either stretching the film perpendicular to the grating directions or along the grating directions. The mechanical flexibility of the PDMS film device was examined on two mechanical stages. We first tested pure gelatine on a PDMS grating film, and the resonator structures were damaged by the surface cracking generated during film stretching. Then we attempted to use different concentration of glycerol to modify the properties of the active layer. As we increased the glycerol concentration gradually from 0% to 5%, 10% and 20%, the surface cracking of the flexible film disappeared. Glycerol wetted PDMS surface and improved the tensile properties of gelatine, preventing the mechanical destruction of the sub-micron scale grating structures. As the glycerol concentration increased, the threshold energy of the laser device also significantly increased, which made it difficult to produce laser output. In the meantime, higher glycerol concentration increased the surface viscosity, and reduced the device stability. Therefore, we optimized the concentration of glycerol in gelatine host as 5% in the PDMS film dye lasers, and used these devices to characterize the wavelength tuning capability.

**Figure 5:**
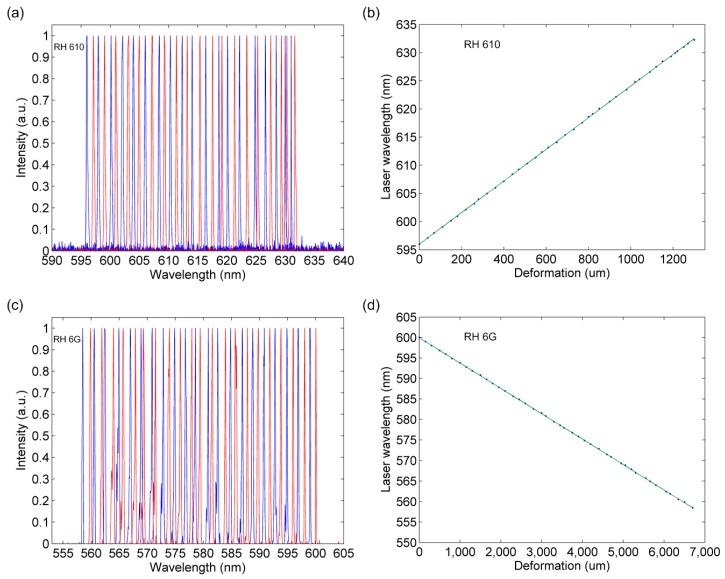
Normalized lasing spectra of DFB film dye lasers in (**a**) perpendicular stretching mode (RH 610) and (**c**) parallel stretching mode (RH 6 G). The relationship of output laser wavelength vs. film deformation in (**b**) perpendicular stretching mode (RH 610) and (**d**) parallel stretching mode (RH 6 G).

To achieve continuous tuning of the laser output, the DFB film dye laser device was fixed on two micrometer stages with 20 mm suspended area for laser pumping, as shown in Figure 1. The mechanical stages could provide high precision control and quantitative measurement of the deformation of the film laser devices. According to the Bragg condition equation, as the grating period increases, the wavelength of the output laser also increases. We have developed two different stretching strategies to change the grating period. In the perpendicular stretching mode, RH 610 (emission spectrum 590–640 nm) was used as the gain medium to match the laser cavity. In this normal stretching scheme, the tuning results of PDMS film lasers were demonstrated in Figure 5a. Different peaks corresponding to different grating periods in the range of 598–632 nm were shown in the figure. The film laser device maintained high quality lasing with 0.12 nm linewidth during the stretching process. Figure 5b shows the relationship between the laser wavelength and film deformation, and the wavelength increased linearly with membrane deformation.

Although compressing the elastomeric film is considered as a common method to decrease the grating period, the control of uniform film compression is quite difficult. Low quality compression of PDMS leads to film distortion, which is unable to realize uniform grating period tuning in a large range. Therefore, we chose a new strategy to achieve grating period reduction by stretching the elastomeric film parallel to the grating. As we stretched the film along the grating direction, the grating period effectively decreased in the active lasing region, and the wavelength shift occurred. In the meantime, the flexible PDMS film maintained surface flatness, thus ensuring lasing production.

For the parallel stretching mode, we chose RH 6 G (emission spectrum 550–600 nm) to match the short-wavelength tuning range. The tunable laser maintained a single mode operation with 0.12 nm linewidth in the range of 557–600 nm, as shown in the lasing spectra in Figure 5c. In Figure 5d, we observed that as the PDMS film was stretched, the output laser wavelength decreased linearly in a large range of nearly 40 nm. Since the grating period reduction is indirectly achieved by film stretching along the grating in this parallel mode, the total PDMS film deformation (6.7 mm) is much higher than the film deformation in the perpendicular mode (1.2 mm). The flexible PDMS has a large deformation capability, allowing the film to be elongated to over 120%. However, only 6% and 35% deformation of the film were used in the perpendicular stretching and parallel stretching experiments, respectively. For the second-order surface emitting laser, although the grating period could be adjusted in a large range, the tuning capability was limited by the laser dye with fixed gain bandwidth. By blending grating structures with different cavity designs and the right choice of laser dyes, a continuously tunable laser device with a wider tuning range could be achieved.

## 4. Conclusions

We have successfully demonstrated a two-directional tuning method of DFB film dye laser devices. The tunable laser device was constructed by a flexible PDMS film and gelatine matrix mixed with glycerol, and surface emitting lasing with low pump thresholds was observed with gain media RH 6 G and RH 610. The laser tuning is simply realized by stretching the PDMS film in two perpendicular directions, and output laser maintained high quality single mode operation. A nearly 40 nm tuning range in each direction could be achieved, covering a large spectrum from 557 to 632 nm. This flexible PDMS film dye laser device has a quick linear response during the tuning process, which suggests strong potential in high precision sensing applications. Moreover, our solid-state dye laser devices offer the convenience of uncomplicated fabrication and stand-alone operation, which can be easily integrated in the optofluidic system, and serve as the coherent light sources for various sensing and spectroscopy applications.
